# Hydrogen Sulfide Upregulates Acid-sensing Ion Channels *via* the MAPK-Erk1/2 Signaling Pathway

**DOI:** 10.1093/function/zqab007

**Published:** 2021-02-19

**Authors:** Zhong Peng, Stephan Kellenberger

**Affiliations:** Department of Biomedical Sciences, University of Lausanne, Rue du Bugnon 27, 1011 Lausanne, Switzerland

**Keywords:** hydrogen sulfide, ASIC, MAPK, p-Erk1/2, regulation, patch-clamp

## Abstract

Hydrogen sulfide (H_2_S) emerged recently as a new gasotransmitter and was shown to exert cellular effects by interacting with proteins, among them many ion channels. Acid-sensing ion channels (ASICs) are neuronal voltage-insensitive Na^+^ channels activated by extracellular protons. ASICs are involved in many physiological and pathological processes, such as fear conditioning, pain sensation, and seizures. We characterize here the regulation of ASICs by H_2_S. In transfected mammalian cells, the H_2_S donor NaHS increased the acid-induced ASIC1a peak currents in a time- and concentration-dependent manner. Similarly, NaHS potentiated also the acid-induced currents of ASIC1b, ASIC2a, and ASIC3. An upregulation induced by the H_2_S donors NaHS and GYY4137 was also observed with the endogenous ASIC currents of cultured hypothalamus neurons. In parallel with the effect on function, the total and plasma membrane expression of ASIC1a was increased by GYY4137, as determined in cultured cortical neurons. H_2_S also enhanced the phosphorylation of the extracellular signal‐regulated kinase (pErk1/2), which belongs to the family of mitogen-activated protein kinases (MAPKs). Pharmacological blockade of the MAPK signaling pathway prevented the GYY4137-induced increase of ASIC function and expression, indicating that this pathway is required for ASIC regulation by H_2_S. Our study demonstrates that H_2_S regulates ASIC expression and function, and identifies the involved signaling mechanism. Since H_2_S shares several roles with ASICs, as for example facilitation of learning and memory, protection during seizure activity, and modulation of nociception, it may be possible that H_2_S exerts some of these effects via a regulation of ASIC function.

## Graphical abstract

**Figure:**
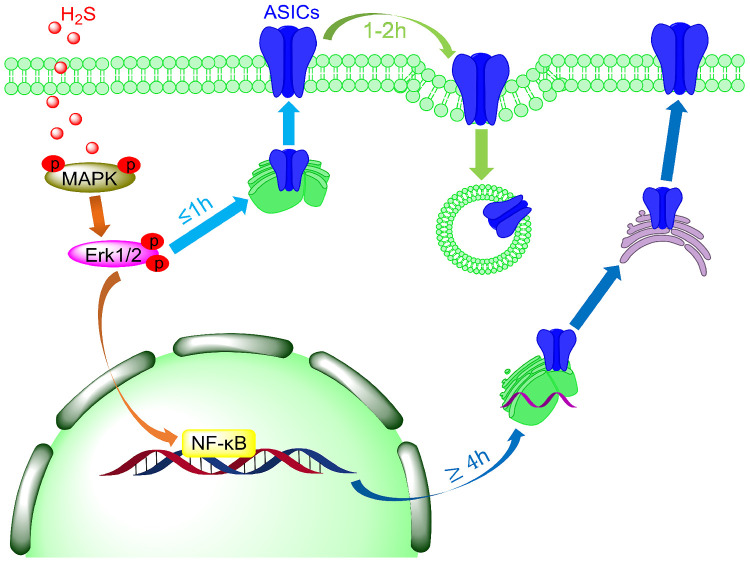

## Introduction

Acid-sensing ion channels^1^^,^^2^ (ASICs) are part of the epithelial sodium channel/degenerin (ENaC/DEG) family.^2^^,^^3^ Four genes encode at least six ASIC subunits (ASIC1a, -1b, -2a, -2b, -3, and -4), which form homotrimeric or heterotrimeric channel complexes. Tissue-dependent differences in ASIC subunit composition due to the different expression patterns of the subunits contribute to a multi-modality of ASIC functions. ASICs are Na^+^-selective^4^ and have in addition a small Ca^2+^ permeability.^1^^,^^5^^,^^6^ Their activation leads therefore generally to excitation of neurons.^7–9^ ASIC1a is distributed throughout the central and peripheral nervous systems, participating in synaptic transmission and plasticity.^10–12^ Dysfunction of ASIC1a is associated with the development of diverse neurological diseases, including neurodegeneration after ischemic stroke,^13–16^ epileptic seizures,^17^ and neurodegenerative diseases.^18^^,^^19^ ASIC3 is widely expressed in peripheral sensory neurons and to some extent in non-neuronal tissues. It is implicated in multimodal sensory perception,^2^^,^^20^ including nociception,^21–23^ mechanosensation,^24^ and chemosensation.^25^^,^^26^

Exposure of ASICs to an acidic pH induces rapid channel opening, followed by desensitization. ASIC3 and some heteromeric ASICs display a sustained current component that follows the transient component. The Texas coral snake toxin MitTx-α/β activates ASIC channels.^27^ The synthetic compound 2-guainidinie-4-methylquinazoline (GMQ) causes persistent activation of ASIC3 at pH 7.4,^28^ while it modulates the activity of other ASICs.^29^ ASIC activity is regulated by many modulators, such as ions, neuropeptides, and animal toxins (reviewed in Wemmie et al.^2^ and Kellenberger and Schild^30^). Reducing reagents potentiate ASIC currents of CNS neurons reversibly and increase acid-induced membrane depolarization, while oxidizing reagents inhibit ASIC currents and reduce acid-induced membrane depolarization.^31^^,^^32^ The endogenous gasotransmitter nitric oxide (NO) also has a direct potentiation effect on ASICs through oxidization of Cys residues.^33^

Recently, hydrogen sulfide (H_2_S) has emerged as the third gasotransmitter after NO and carbon monoxide. H_2_S had long been known as a toxic agent. Several studies have shown that H_2_S is produced in several organs/tissues of our body, as in the nervous,^34^ digestive,^35^ and endocrine system.^36^ Endogenous H_2_S is produced by three pyridoxal-5′-phosphate-dependent enzymes: (1) cystathionine-β-synthase (CBS, EC 4.2.1.22) and (2) cystathionine-γ-lyase (CSE, EC 4.4.1.1)^37–39^—which both produce H_2_S directly, and (3) 3-mercaptopyruvate sulfurtransferase (3MST, EC 2.8.1.2),^40^ which produces H_2_S indirectly. In mammals, CBS is predominantly expressed in the brain, while CSE and 3MST are found mostly outside the brain.^41–43^ Physiological concentrations of H_2_S, generated by CBS and CSE, of 10–100 μM in the blood were reported,^44^^,^^45^ but the accurate concentration of free H_2_S remains difficult to determine. H_2_S regulates ion channels, similarly to other gasotransmitters, through modulation of Cys residues by the formation of persulfide (-SSH) bonds. This modification has been termed protein S-sulfuration.^46^ H_2_S was shown to potentiate or activate glutamate receptors and ATP-sensitive K^+^ channels,^47–49^ and inhibit Ca^2+^ currents in native pancreatic β-cells,^50^ leading to reduced glucose-stimulated insulin secretion.

Here, we report that H_2_S enhances ASIC currents as well as total and cell surface expression in cultured brain neurons, and we provide evidence that this regulation involves the mitogen-activated protein kinase (MAPK)–extracellular signal-regulated kinase (Erk)1/2 signaling pathway.

## Materials and Methods

### Ethical Approval

All animal handling procedures were done in accordance with institutional and Swiss guidelines and approved by the authorities of the Canton of Vaud. All animal experiments respected the Swiss Animal Welfare legislation and were reviewed by the Veterinary Service of the Canton de Vaud (Animal Welfare Act 2019; Project License N° 1750.4 licensed to Dr. Stephan Kellenberger).

### Recombinant Expression of ASICs in CHO Cells

The cDNAs used for heterologous expression of ASIC channels were as follows: human ASIC1a, GenBank ID: U78181; rat ASIC1b, GenBank ID: 3445467; human ASIC2a, GenBank ID: U57352; rat ASIC3, GenBank ID: 27465600. The ASIC1a-C466A-C471A-C497A-C528stop cDNA construct^51^ was kindly provided by Miguel van Bemmelen (University of Lausanne, Switzerland). The experiments with recombinant human ASIC1a in the present study were carried out with the ASIC1a clone containing the mutation G212D, whose main effect is an acceleration of the desensitization kinetics.^52^ All constructs were expressed in Chinese hamster ovary (CHO) cells. The transient transfection of CHO cells was performed as reported previously.^52^ In brief, CHO cells were cultured at 37°C in a humidified atmosphere with 5% (v/v) CO_2_, and passaged twice a week. CHO cells were transiently co-transfected with ASIC and EGFP cDNA, using Rotifect transfection reagent (Carl Roth, D-Karlsruhe). CHO cells were cultured in Ham’s F-12K (Kaighn’s) medium (ThermoFisher Scientific) supplemented with 10% (v/v) fetal bovine serum (FBS, ThermoFisher Scientific) and 1% penicillin–streptomycin (5000 U·mL^-1^, ThermoFisher Scientific). Electrophysiological measurements were performed 24–48 h after transfection.

### Embryonic Mouse Cerebral Cortex and Hypothalamus Neuron Culture

Twenty-four pregnant mice and 144 mouse embryos were used in these experiments to obtain cells for culture; ASIC1a^−/−^ mice (C57BL/6 background) were provided by Dr. John Wemmie (University of Iowa). Mice used in the experiments were kept in the departmental animal house and maintained on a 12 h light/dark cycle with food and water *ad libitum*. Neuron culture was performed as previously described.^53^ Briefly, Days 14–15 pregnant mice were sacrificed by exposure to CO_2_, the embryos were killed, and the cortex and hypothalamus of the E14-15 embryos were dissected in ice-cold HBSS medium (ThermoFisher). Brain tissues were chopped into small pieces (∼1 mm) and incubated at 37°C for 18 min in 0.05% Trypsin-EDTA (ThermoFisher), then washed 3 times in Neurobasal medium (ThermoFisher) containing 10% FBS, and dissociated into single cells. After a 5-min centrifugation at 1000 rpm, neurons were resuspended in Neurobasal/FBS medium. For the biochemical assay, neurons were seeded at 300 000 cells/dish on 60-mm Petri dishes previously coated with poly-L-lysine. For electrophysical recording, neurons were seeded at 50 000 cells/dish on 35-mm Petri dishes containing three 15-mm diameter glass coverslips previously coated with poly-L-lysine. For both 60-mm dishes and coverslips, the medium was replaced after 12 h by Neurobasal Medium Electro (ThermoFisher) containing the B27 serum-free supplement, the GlutaMAX supplement (ThermoFisher), and Gentamicin (10 µg·mL^-1^ final concentration, ThermoFisher). Neuronal cultures were maintained at 37°C in a humidified atmosphere with 5% (v/v) CO_2_, and every 2–3 days, half of the medium was replaced with fresh plating medium. Patch-clamp experiments of hypothalamus neurons were carried out after at least 12 days after seeding. Biochemical experiments of cortical neurons were done after at least 9 days after seeding.

### Electrophysiological Recording

Electrophysiological recordings were done using the whole-cell patch-clamp technique in voltage-clamp mode with an EPC10 patch-clamp amplifier (HEKA Elektronik-Harvard Bioscience) as previously described.^53^ The solution exchange was carried out using computer-controlled electrovalves (cF-8VS) and the MPRE8 perfusion head (Cell MicroControls, Norfolk, VA). Data were acquired with Patchmaster software and analysis of the currents was carried out with Fitmaster (HEKA Elektronik-Harvard Bioscience). The sampling interval and the low-pass filtering were set to 50 µs and to 3 kHz, respectively.

The pipette solution contained, in mM, 120 KCl, 30 NaCl, 10 HEPES, 5 EGTA, 2 MgATP, 1 MgCl_2_ and 0.5 CaCl_2_, adjusted to pH 7.2 with Tris-base. The osmolarity of the pipette solution was 280–300 mOsm (Advanced Instrument Osmometer, Norwood, MA, USA). The extracellular Tyrode solution contained, in mM, 140 NaCl, 5 KCl, 10 glucose, 2 CaCl_2_, and 1 MgCl_2_, buffered to various pH values with either 10 mM HEPES (pH > 6.0) or 10 mM 2-(N-morpholino)-ethanesulfonic acid (MES; pH ≤ 6.0). The osmolarity of the extracellular solution was 310–320 mOsm. The pH of the solutions was controlled on the day of the experiment and adjusted if necessary. All recordings were performed at room temperature (23 ± 2°C).

### Plasma Membrane Protein Extraction

Plasma membrane protein extraction was carried out as previously described.^54^ Briefly, the cultured cortical neurons were washed 3 times with ice-cold PBS, and the neurons were harvested with a cell scraper. After centrifugation at 500*g* for 5 min at 4°C, the pellet was resuspended in ice-cold homogenization buffer (250 mM sucrose, 1 mM EDTA, 10 mM Tris-HCl buffer, pH 7.2, containing 1:100 diluted protease and phosphatase inhibitor cocktail, Sigma Aldrich). The neurons were then sonicated 15 s using a probe sonicator (Bandelin SONOPULS HD 2200), centrifuged at 10 000*g* for 10 min at 4°C, and the supernatant was collected. The sonication and supernatant extraction was done twice. The supernatant was centrifuged at 25 000*g* in an Optima MAX-XP benchtop ultracentrifuge with an MLA-55 rotor (Beckman Coulter Inc., Brea, CA) for 1 h at 4°C and the pellet was collected and resuspended in starting buffer (225 mM mannitol, 75 mM sucrose, and 30 mM Tris-HCl, pH 7.4). The suspension was centrifuged for 20 min at 25 000*g*, and the pellet was collected and lysed with 1× sample loading buffer (0.3 M sucrose, 2% SDS, 2.5 mM EDTA, 60 mM Tris, pH 8.8, 0.05% (w/v) bromophenol blue, 25 mM DTT), followed by heating at 95°C for 10 min.

### Biochemical Assay

Western blot analysis was carried out as previously described.^52^ Briefly, 10 µL protein samples were separated on 10% SDS-PAGE gels for 2 h electrophoresis at 100 V, then transferred to 0.2 µM nitrocellulose membranes (Amersham Biosciences) at 4°C, 100 V for 2 h. After the transfer, the blot was blocked with 5% milk (in TBST buffer, Tris-buffered saline with 0.1% Tween 20 solution) for 1 h at room temperature, followed by 2% BSA in TBST buffer at room temperature. The blot was incubated at 4°C overnight with the primary antibodies, followed, after washing, by the HRP-labeled secondary antibody for 2 h at room temperature. The signals were detected using the Fusion SOLO chemiluminescence system (Vilber Lourmat, Marne-la-Vallée, France) using SuperSignal™ West Femto Maximum Sensitivity Substrate (Thermo Scientific). The following antibodies were used: anti-ASIC1 (1:1000, rabbit,^10^ kindly provided by Dr John Wemmie), Anti-Actin (1:1000, rabbit; A2066, Sigma Aldrich), anti-Na^+^/K^+^ ATPase α1 (1:10 000, rabbit,^55^ kindly provided Dr Käthi Geering), anti-Erk1/2 (1:500, rabbit; 4695S, Cell Signaling), anti-phosphor-ERK1/2 (1:200, mouse; 9106S, Cell Signaling), anti-JNK (1:500, rabbit; 9252S, Cell Signaling), anti-phosphor-JNK (1:500, rabbit; 4668s, Cell Signaling), anti-p38 (1:500, rabbit; 9211s, Cell Signaling), anti-phosphor-p38 (1:500, rabbit; 9212s, Cell Signaling), donkey anti-rabbit IgG (1:2000; NA934VS, GE Healthcare), and rabbit anti-mouse IgG (1:2000; 06-371, Sigma Aldrich). p38 and JNK expression levels were determined using the anti-p38 or anti-JNK antibody after stripping of the p-p38 or p-JNK membrane. The blot membrane was incubated in stripping buffer (0.15% glycine, w/v; 0.1% SDS, w/v; 1% Tween 20, v/v; pH adjusted to 2.2 with HCl) at RT for 2 times 5 min, and washed 2 times for 5 min in TBST. Afterward, the membrane was re-blocked. Quantification was done using ImageJ. β-actin was used as the total protein control, and Na^+^/K^+^ ATPase α1 as plasma membrane protein control, to which the band signals were normalized.

### Reagents

All drugs were purchased from Sigma-Aldrich (Buchs, Switzerland) unless otherwise mentioned.

### Data Analysis and Statistics

Results are expressed as the mean ± SEM. Statistical comparisons were performed using Student’s *t*-test for comparison between two groups or for paired comparisons, and one-way ANOVA followed by Dunnett’s *post hoc* test when more than two groups were involved. For the analysis of the (time or concentration) series of the biochemical experiments (non-Gaussian distribution), Multiple Mann–Whitney tests were used. Statistical tests were carried out with Graphpad Prism8 (GraphPad, San Diego). The sustained ASIC3 currents were measured during the last 2 s of the acidic pH application.

## Results

### H_2_S Potentiates ASIC1a Currents in a Concentration- and Time-dependent Way

**Figure 1.**
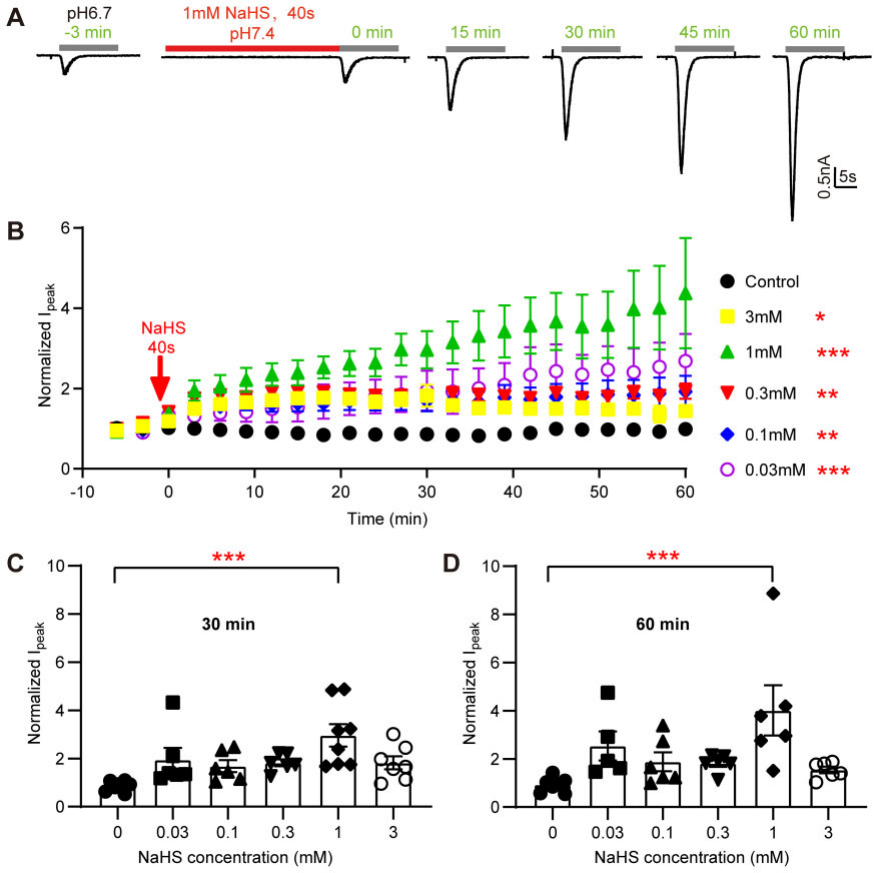
The H_2_S Donor NaHS Potentiates ASIC1a Currents in a Concentration- and Time-Dependent Manner. (**A**) Representative current traces obtained with whole-cell patch-clamp of human ASIC1a-expressing CHO cells at −60 mV, induced by acidification to pH 6.7 at different time points as indicated. One millimolar NaHS was administered once in the experiment for 40 s (red horizontal line). (**B**) pH 6.7-induced ASIC1a peak current amplitudes (Mean ± SEM) measured over a period of 60 min without NaHS (control, black symbols) or with a 40-s exposure at the indicated concentration just before the time point 0. The current amplitudes were normalized to the pH 6.7-induced currents measured before the NaHS exposure (at −3 and −6 min), *n* = 5–7. *P < 0.05; **P < 0.01; ***P < 0.001; compared with the control (black symbols) over the period 0–60 min by one-way ANOVA test and Dunnett’s *post hoc* test. **C**, **D**, pH 6.7-induced peak current amplitudes of ASIC1a expressed in CHO cells at 30 min (**C**) and 60 min (**D**) after 40-s exposure to the indicated concentration of NaHS, from the experiments shown in (B), normalized to the pH 6.7-induced current amplitude before NaHS exposure, *n* = 5–6. The bar and error bars indicate mean±SEM. ***P < 0.001, compared to control, by one-way ANOVA test and Dunnett’s *post hoc* test.

Currents of ASIC1a, heterologously expressed in CHO cells, were recorded by whole-cell patch-clamp. Since the gas H_2_S is difficult to dissolve in aqueous solutions, and its concentration would be hard to control, the H_2_S donor NaHS was used, which releases H_2_S rapidly.^56^ ASIC1a was activated every 3 min by a 10-s acidification from pH 7.4 to 6.7. The acidification induced a rapidly developing transient inward current (Figure 1A). When applied alone, 1 mM NaHS did not generate any current in ASIC1a-expressing CHO cells (Figure 1A). However, the pH 6.7-induced ASIC1a current amplitude increased after a 40-s incubation with 1 mM NaHS. One hour after the short NaHS exposure, without any additional administration of NaHS, the ASIC1a current was increased by 4.4 ± 1.3-fold (mean±SEM, *n* = 6; Figure 1A and B). To further characterize the effects of H_2_S on acid-induced ASIC1a activation, the changes in ASIC1a current amplitudes over time were also determined after a 40 s exposure to other concentrations of NaHS (Figure 1B). With concentrations of 30 µM to 3 mM NaHS, the increase in ASIC1a current was statistically significant if analyzed for the duration of the experiment. A concentration of 30 μM H_2_S can be attained under multiple physiological and pathological conditions.^57^^,^^58^ Although no gradual concentration dependence of the NaHS effect was observed, it is obvious from the time course and from the comparison after 30 and 60 min (Figure 1C and D) that the current activation with 3 mM NaHS was smaller than that observed with 1 mM (*P* = 0.0026 at 60 min). With 3 mM NaHS, a tendency of a maximal potentiation in the time window of 12–30 min was observed, before it gradually decreased (Figure 1B). At the physiological pH 7.4 and a temperature of 20°C, approximately 70% of the total sulfide exists as the HS^−^, which, via prior formation of intermediate species such as polysulfides, can form covalent persulfide bonds with Cys.^59^

### NaHS Potentiates the Current of all Tested ASIC Isoforms

**Figure 2.**
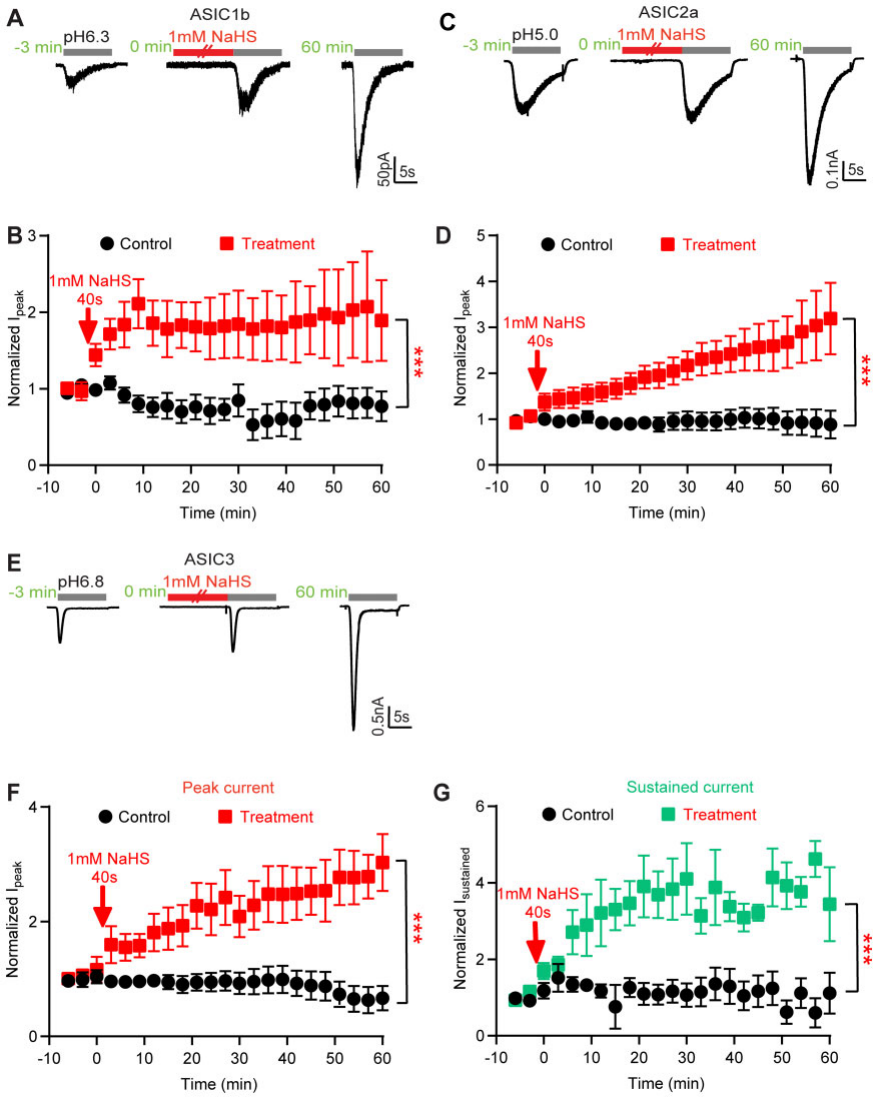
NaHS Potentiates the Current of All Tested ASIC Isoforms. Current traces and data were obtained by whole-cell patch-clamp at −60 mV of CHO cells transfected with the indicated ASIC isoforms. The indicated time points are relative to the 40-s application of 1 mM NaHS. All quantified currents had been normalized to that induced by acid before the NaHS treatment (at −3 and −6 min). The statistical significance in (**B**), (**D**), (**F**), and (**G**) is based in each case on a comparison between treatment and control over the period 0–60 min by one-way ANOVA test and Dunnett’s *post hoc* test; ***P < 0.001. (**A**) Representative rat ASIC1b current traces, induced by acidification to pH 6.3 at different time points, as indicated. (**B**) Time course of pH 6.3-induced peak ASIC1b current amplitudes measured without (control, black symbols) or with a 40-s exposure to 1 mM NaHS as indicated (treatment, red symbols), *n* = 5. (**C**) Representative human ASIC2a current traces, induced by acidification to pH 5.0. (**D**) Time course of pH 5.0-induced ASIC2a peak current amplitudes measured without (control, black symbols) or with a 40-s exposure to 1mM NaHS as indicated (treatment, red symbols), *n* = 5–6. (**E**) Representative rat ASIC3 current traces, induced by acidification to pH 6.8 at different time points, as indicated. (**F**) Time course of pH 6.8-induced ASIC3 peak current amplitudes measured without (black symbols) or with a 40-s exposure to 1 mM NaHS as indicated (red symbols), *n* = 5–6. (**G**) Time course of pH 6.8-induced ASIC3 sustained current amplitudes measured without (black symbols) or with a 40-s exposure to 1 mM NaHS (green symbols) as indicated, *n* = 5–6.

Next, it was tested whether NaHS modulates other ASIC isoforms. The measurements of ASICs transiently expressed in CHO cells were carried out by repetitive stimulation at a pH that induced approximately 20% of the maximal peak current amplitude (pH 6.3 for ASIC1b, pH 5.0 for ASIC2a, pH 6.8 for ASIC3). To test whether NaHS can regulate ASICs, NaHS was applied at a concentration of 1 mM once during 40 s, as described for ASIC1a. After 60 min, the NaHS-induced current increase amounted to ∼2-fold with ASIC1b, and ∼3-fold with ASIC2a (Figure 2A–D). Furthermore, NaHS enhanced the ASIC3 peak current by ∼3-fold, and its sustained current by ∼4-fold (Figure 2E–G). These results indicate that H_2_S potentiates all functional homomeric ASICs. The analysis of two selected time points, 0 min (thus directly after NaHS exposure) and 60 min, showed a significant potentiation for all isoforms except ASIC2a at 0 min, and for ASIC1a and ASIC3 at 60 min (Supplementary Figure S1A and B, multiple Mann–Whitney tests between the 1 mM NaHS condition and the respective control experiment).

### The ASIC1a Current Potentiation by NaHS is Not Due to a Change in pH Dependence

**Figure 3.**
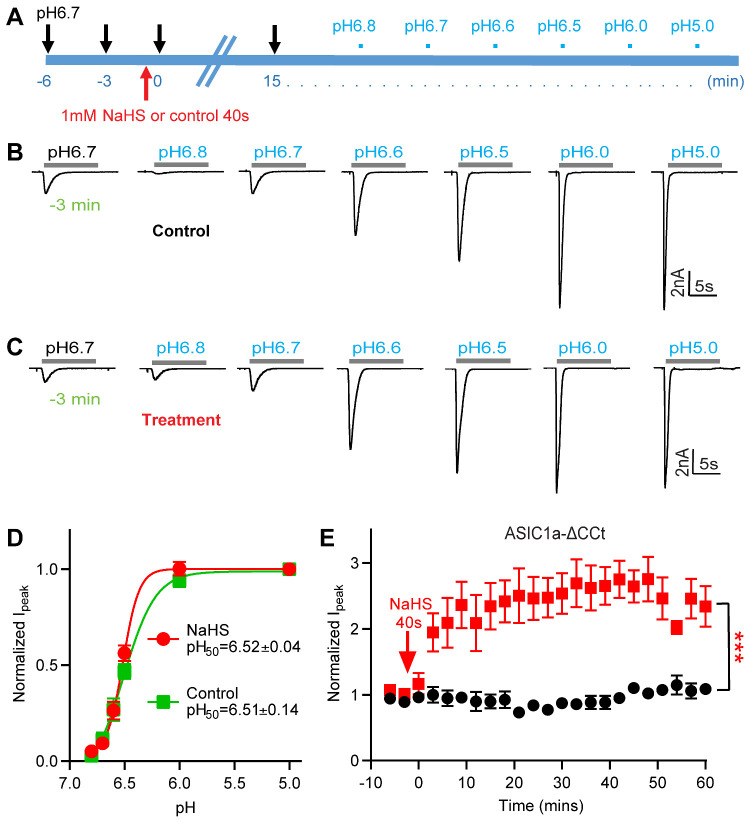
NaHS Potentiation of ASIC1a Currents Is Not Due to a Change in pH Dependence. (**A**) Schematic representation of the protocol used to test whether the exposure to 1 mM NaHS induces a shift in the pH dependence of ASIC1a expressed in CHO cells. (**B** and **C**) Representative ASIC1a current traces for the construction of a pH–response curve. Fifteen minutes before starting the recording of the pH-response curve, the cell was exposed during 40 s to a control solution (**B**, “control”) or to a solution containing 1mM NaHS (**C**, “treatment”). (**D**) ASIC1a peak current amplitudes, normalized to the peak amplitude induced by pH 5.0, for cells exposed to 1 mM NaHS (treatment, red) or not (control, green), *n* = 5–9. The solid lines represent a fit to the Hill equation. The pH_50_ values were not different between the two conditions (unpaired Student’s *t*-test). (**E**) Time course of the pH 6.7-induced current of CHO cells expressing a mutant ASIC1a in which the intracellular C-terminal Cys residues were mutated or deleted (ASIC1a-C466A-C471A-C497A-C528stop, ASIC1a-ΔCCt), measured without (control, black symbols) or with a 40-s exposure to 1 mM NaHS at time point 0, as indicated (treatment, red symbols), *n* = 4–6. ***P < 0.001, comparison between treatment and control over the period 0–60 min by one-way ANOVA test and Dunnett’s *post hoc* test. Current amplitudes were normalized to the pH 6.7-induced current amplitude measured before NaHS exposure (at −3 and −6 min).

To determine whether the observed potentiation of pH 6.7-induced currents by NaHS is due to a shift in the pH dependence, the pH dependence of ASIC1a activation was determined in ASIC1a-expressing CHO cells 15 min after a 40-s exposure to NaHS or control solution (Figure 3A). NaHS treatment did not affect the pH of half-maximal activation (pH_50_; 6.51 ± 0.14 for control, 6.52 ± 0.04 for NaHS treatment, *n* = 5–9; Figure 3B–D). Consistent with Figures 1A–B and 3B–C, the pH 6.7-induced current was significantly increased at 15 min by exposure to NaHS but not by exposure to control solution, and the ASIC1a current increase was significantly different between these two conditions (Supplementary Figure S2). This finding indicates that the potentiation of the ASIC currents by H_2_S does not depend on an increase of the apparent affinity of ASICs to acid.

### C-Terminal Cys Residues are Not Involved in the NaHS Modulation

Previous studies have suggested in various ion channels an involvement of Cys residues in H_2_S modulation.^60^^,^^61^ According to the structural information,^62^ human ASIC1a contains only one unpaired extracellular Cys residue, Cys275. A recent study observed a transient potentiation of ASIC currents by NaHS. In the cited study it was shown that extracellular pre-treatment with the hydrophilic sulfhydryl reagent sodium (2-sulfonatoethyl) methanethiosulfonate did not prevent this current modulation, and it was concluded that the effect is not mediated by extracellular Cys residues.^63^ ASIC1a contains in addition Cys residues in the transmembrane and cytoplasmic parts. Since the intracellular C-terminus of ASIC1a contains many Cys residues that may affect ASIC function, NaHS modulation was examined on the mutant ASIC1a C466A/C471A/C497A/C528stop (ASIC1a-ΔCCt), in which the C-terminal Cys residues were eliminated by mutation and truncation.^51^ The functional properties of ASIC1a-ΔCCt had been shown to be very similar to those of WT.^51^ In ASIC1a-ΔCCt, NaHS also induced a robust, time-dependent potentiation over time (Figure 3E). In the WT, the control response at the first stimulation after NaHS exposure was increased by 38 ± 9% (mean ± SEM, relative to the average of the control responses at −6 and −3 min). This increase was different from that of the control experiments without NaHS (*P* = 0.002, unpaired Student’s *t*-test) in the WT (Figure 1C, *n* = 7), while the increase of 12 ± 13% (mean ± SEM) in the ASIC1a-ΔCCt mutant was statistically not different from the corresponding control (*P* = 0.119, *n* = 5). Although this shows an apparent difference directly after NaHS exposure between the mutant and WT ASIC1a, this difference was not statistically significant. When observed over the duration of the experiment, the potentiation of the ASIC1a-ΔCCt mutant by NaHS was indistinguishable from that of ASIC1a WT. This indicates that C-terminal Cys residues of ASIC1a are not involved in NaHS-induced potentiation, and suggests together with the previous observations a possible indirect effect of H_2_S on ASICs.

### H_2_S Donors Potentiate Endogenous Acid-Induced ASIC Currents in Cultured Hypothalamus Neurons

**Figure 4.**
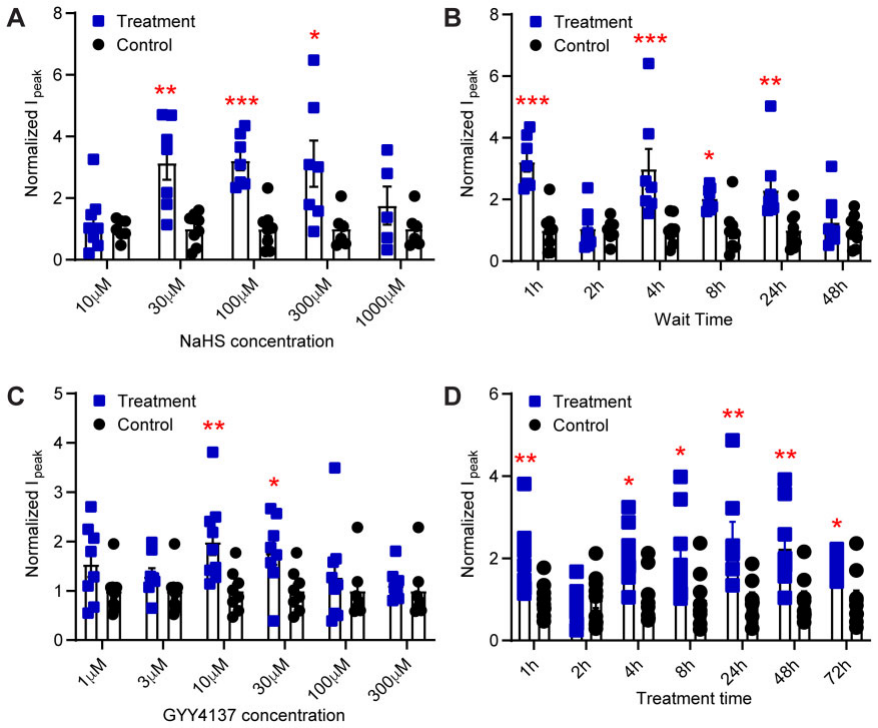
H_2_S Donors Potentiate Endogenous ASIC Currents in Cultured Mouse Hypothalamus Neurons. The currents were measured by whole-cell voltage-clamp at −60 mV from cultured hypothalamus neurons of mice. The bars and error bars indicate mean±SEM. Together with each treatment condition, a number of control cells (ie, treatment protocol with solution lacking the H_2_S donor) were measured, and the current amplitudes obtained for the treatment and for the respective control were normalized to the average of the control. (**A** and **B**) Cells were exposed for 1 min to NaHS and then put back into the incubator for a defined period before the current measurement. (A) pH 6.6-induced current amplitudes measured 1 h after exposure to the indicated NaHS concentration (treatment, blue) or to control solution without NaHS (control, black), *n* = 5–8. **B**, pH 6.6-induced current amplitudes measured at the indicated time after a 1-min 100 µM NaHS (blue symbols) or control exposure (black symbols), *n* = 6–9. (**C** and **D**) The H_2_S donor GYY4137 at the indicated final concentrations was added to the culture medium, and cells were incubated in the cell incubator for the indicated period. (C) pH 6.6-induced current amplitudes measured after 1 h incubation with the indicated concentration of GYY4137 (treatment, blue) or with the addition of solution lacking GYY4137 (control, black), *n* = 7–9. (D) pH 6.6-induced current amplitudes measured after incubation for the indicated time with 10 µM GYY4137 (treatment, blue) or with solution lacking GYY4137 (control, black), *n* = 8–10. *P < 0.05; **P < 0.01; ***P < 0.001; comparison of each treatment condition with the corresponding control condition by unpaired multiple Mann–Whitney tests.

To gain insights into the regulation of neuronal ASICs by H_2_S, the effect of NaHS on acid-induced currents in primary cultures of hypothalamus neurons was tested. ASIC currents in central neurons, activated by pH ≥ 6 are due to ASIC1a homotrimers or heterotrimers involving ASIC1a, -2a, and -2b.^10^^,^^64^ Several studies have reported transient acidification-induced currents in rodent hypothalamus neurons that were identified as ASIC currents based on their biophysical and pharmacological properties.^65–67^ In our hands, exposure of cultured hypothalamus neurons to pH 6.6 induced rapid, desensitizing inward currents consistent with ASIC activity (Supplementary Figure S3). With the cultured neurons, stable recordings over 1 h, as done in Figure 1 with the transfected CHO cells, were not possible. Therefore, a different strategy was used. Dishes with cultured neurons were removed from the incubator and incubated for 1 min in the recording solution with or without NaHS, before this solution was replaced by culture medium and the cells were put back in the incubator. In the first set of experiments, cells were exposed to different concentrations of NaHS, and currents were measured after 1 h exposure in the incubator. NaHS potentiated the pH 6.6-induced ASIC currents in a concentration-dependent way **(**Figure 4A). Strikingly, the effect of NaHS (10 µM–1 mM) was biphasic, with concentrations of 30–300 µM increasing ASIC currents, whereas lower (10 µM) or higher concentrations of NaHS (1 mM) did not increase the currents (Figure 4A). To determine the time dependence of ASIC modulation by NaHS in cultured hypothalamus neurons, acid-induced currents were recorded at different time points after a 1-min 100 µM NaHS exposure. An increase of ASIC currents was observed in the time span between 1 and 24 h, not however at 2 h after the NaHS treatment (Figure 4B). The NaHS modulation of pH 6.6-induced ASIC currents was lost 48 h after the NaHS incubation.

NaHS releases H_2_S rapidly, and oxidation and reaction with other agents in the water reduce the actual concentration of H_2_S in solution promptly.^68^ In pathological conditions, the increase of endogenous H_2_S levels can last a long time.^69^ A different H_2_S donor, GYY4137, has been shown to release H_2_S slowly, over a period of hours to days, both in aqueous media and when administered to living animals.^56^ To investigate the ASIC modulation by H_2_S in cultured hypothalamus neurons over a longer time period and with a different H_2_S donor, GYY4137 was added to the cultures in the incubator, and left until the time of current measurement. In the first series of experiments, the neurons were exposed to different GYY4137 concentrations for 1 h before the measurement of the current amplitude. As control, the same volume of culture medium, but without GYY4137, was added to the culture dish, and currents were measured after 1 h of incubation. Similar to NaHS, GYY4137 potentiated the pH 6.6-induced currents in a concentration-dependent manner (Figure 4C). A statistically significant current increase was measured at 10 and 30 µM, whereas lower or higher concentrations (≤3 or ≥100 µM) did not increase the ASIC currents (Figure 4C). The time course of the GYY4137 effect, measured at a concentration of 10 µM, shows potentiation of the pH 6.6-induced currents by GYY4137 at all tested time points except after 2 h (Figure 4D).

### H_2_S Regulates the Expression of ASIC1a and Activates the Erk1/2 Signaling Pathway

**Figure 5.**
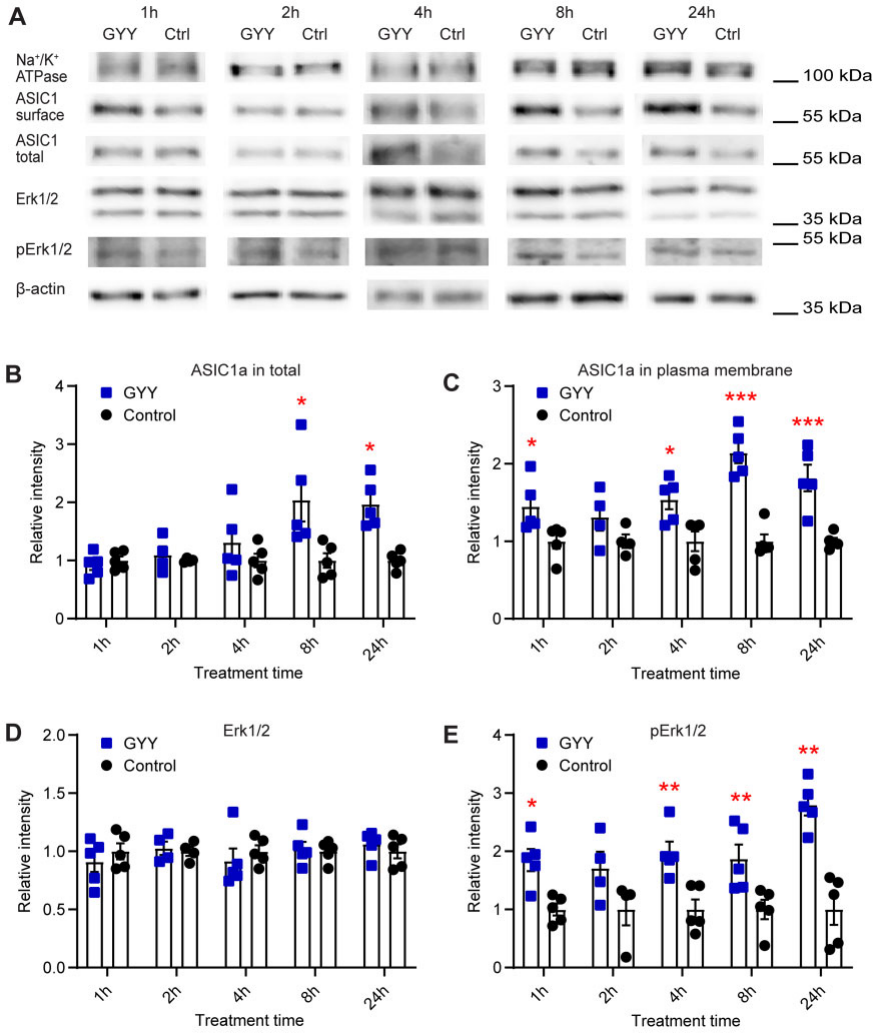
H_2_S Donors Regulate the Expression of ASIC1a and the Activation of the Erk1/2 Signaling Pathway. The biochemical experiments were carried out in cultured mouse cortical neurons. Total and plasma membrane proteins were isolated, separated on SDS-PAGE, and specific proteins were visualized as described in the “Materials and Methods” section. (**A**) Representative Western blots of total and plasma membrane ASIC1a, and Erk1/2, p-Erk1/2, Na^+^/K^+^ATPase, and β-actin as indicated, after incubation with 10 µM GYY4137 (GYY) or without (Ctrl) for the indicated time. β-actin was used as a control for the total protein, and Na^+^/K^+^ ATPase α1 as a control for plasma membrane proteins. The β-actin and Na^+^/K^+^ ATPase bands shown in (A) were from the same sample, but not in all cases from the same lane on the gel, as the bands shown above or below. (**B–E**) Cells were exposed to 10 µM GYY4137 (GYY, blue symbols) or to control medium (control, black symbols) for the indicated time. The measured intensities were normalized to the average intensity of the corresponding control. (**B**) Total expression of ASIC1a, *n* = 4–5. (**C**) Plasma membrane expression of ASIC1a, *n* = 4–5. (**D**) Expression of Erk1/2, *n* = 4–5. (**E**) Expression of p-Erk1/2, *n* = 4–5. *P < 0.05; **P < 0.01; comparison of each treatment condition with the corresponding control condition by multiple Mann–Whitney tests.

To test whether the observed ASIC current increase upon exposure to GYY4137 is due to a changed ASIC expression, the effect of H_2_S on total and plasma membrane expression of ASIC1a was measured. Since the hypothalamus is a small nucleus and many animals would be required to obtain enough cells for a biochemical analysis, ASIC expression was determined in cortical neurons, in which ASIC currents of relatively high amplitude have been measured.^10^^,^^70^ Primary cultures of cortical neurons were incubated for different time periods with 10 µM GYY4137. After extraction of total proteins and of plasma membrane-resident proteins by a centrifugation protocol,^54^^,^^71^^,^^72^ and separation by SDS-PAGE, total and plasma membrane expression of ASIC1a was determined by Western blot analysis (Figure 5A–C). Representative blots indicate an increased ASIC1a expression after GYY4137 exposure (Figure 5A). The increase in total ASIC1a expression appeared only at ≥8 h, but was maintained at the latest time point measured 24 h (Figure 5B). ASIC1a expression at the plasma membrane was significantly increased after GYY4137 treatment for 1 h, and then again at incubation times ≥ 4 h, not however after 2 h (Figure 5C).

A previous study reported H_2_S regulation of ENaC expression, and demonstrated an implication of Erk1/2, an important member of the MAPK cascade, in the regulation of the ENaC expression by H_2_S.^73^ Activation of Erk1/2 (detected as phosphorylated Erk1/2, p-Erk1/2) can regulate the expression of ASIC1a.^74^ It is therefore possible that H_2_S may potentiate ASIC currents *via* the Erk1/2 kinase cascade. For this reason, the expression of Erk1/2 and p-Erk1/2 in cultured cortical neurons after exposure to 10 µM GYY4137 was examined by Western blot. GYY4137 did not significantly change the Erk1/2 expression (Figure 5A and D); it increased however the p-Erk1/2 signal indicating an activation of Erk1/2 (Figure 5E). This increase was statistically significant at all time points except at 2 h.

### H_2_S Does Not Activate the JNK and p38 Pathways in Cortical Neurons

**Figure 6.**
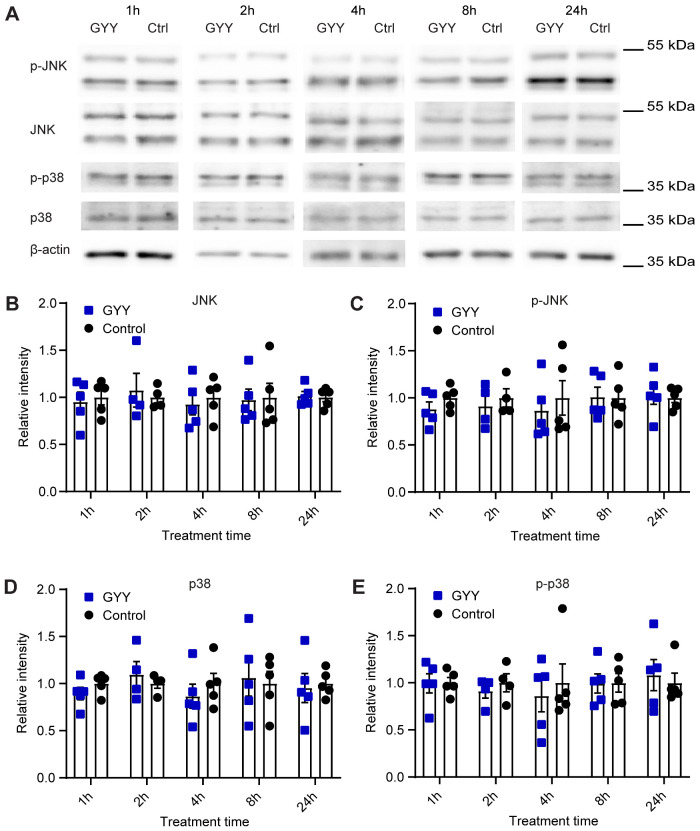
The p38 and JNK Signaling Pathways Are Not Involved in the Upregulation of ASIC1a Expression by H_2_S Donors. The biochemical experiments were carried out in cultured mouse cortical neurons. Total proteins were isolated, separated on SDS-PAGE, and specific proteins were visualized as described in the “Materials and Methods” section. (**A**) Representative Western blots of total JNK, p-JNK, p38, p-p38, and β-actin as indicated, after incubation with 10 µM GYY4137 (GYY) or without (Ctrl) for the indicated times. β-actin was used as control. The β-actin bands shown in (A) were from the same sample, but not in all cases from the same lane on the gel, as the bands shown above. (**B–E**) Cells were exposed to 10 µM GYY4137 (GYY, blue symbols) or to control medium (control, black symbols) for the indicated time. The measured intensities were normalized to the average intensity of the corresponding control. (B) Expression of JNK, *n* = 4–5. (**C**) Expression of p-JNK, *n* = 4–5. (**D**) Expression of p38, *n* = 4–5. (**E**) Expression of p-p38, *n* = 4–5. Comparison of each treatment condition with the corresponding control condition by multiple Mann–Whitney tests indicated no significant differences

MAPKs constitute a large family of protein kinases that respond to a wide range of extracellular stimuli, which lead to phosphorylation of their serine and threonine residues.^75^ Besides Erk1/2, other MAPK subfamilies exist, such as the p38 and c-Jun amino-terminal kinases (JNK). p38 and JNK are activated at the MAPK level by similar types of stimuli. To test whether the upregulation of ASIC1a expression by H_2_S may also depend on other MAPK cascades, expression of the total and of the activated forms of JNK and p38 was determined by Western blot (Figure 6). In cultured cortical neurons, 10 µM GYY4137 did not significantly change the expression of JNK and p-JNK (Figure 6A–C), nor of p38 and p-p38 (Figure 6A, D, and E).

### H_2_S Upregulates ASIC1a Expression *via* the MAPK Signaling Pathway

**Figure 7.**
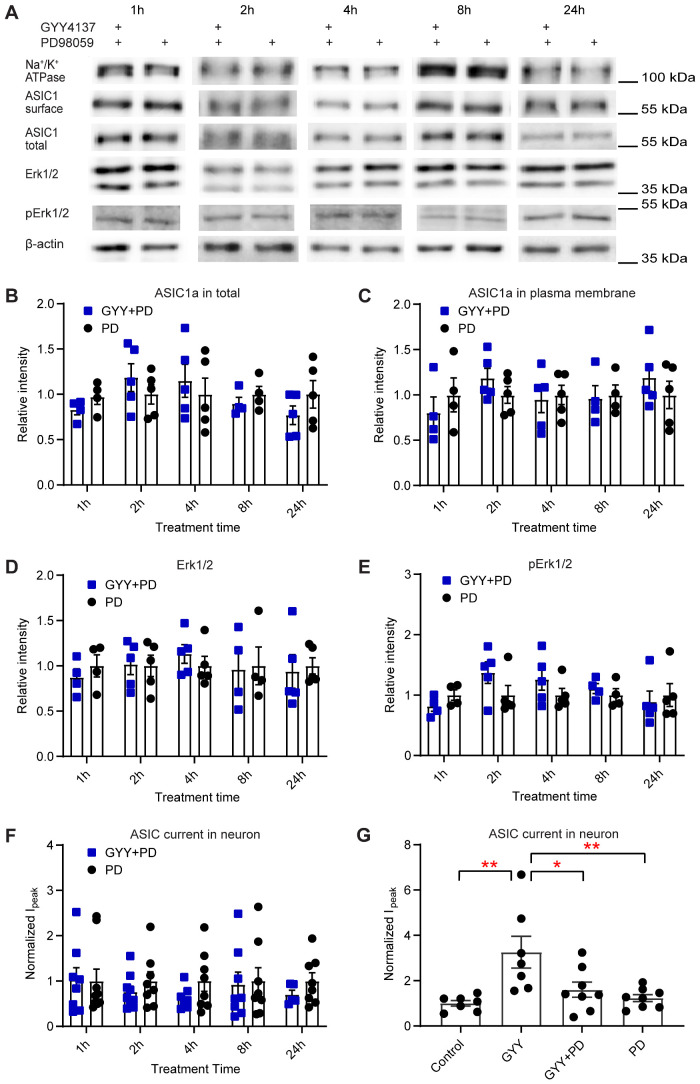
H_2_S Donors Upregulate ASIC1a Expression *via* the MAPK Signaling Pathway. The biochemical experiments were carried out in cultured mouse cortical neurons. Total and plasma membrane proteins were isolated, separated on SDS-PAGE, and specific proteins were visualized as described in “Materials and Methods” section. (**A**) Representative Western blots of total and plasma membrane ASIC1a, and of Erk1/2, p-Erk1/2, Na^+^/K^+^ATPase, and β-actin after incubation for the indicated times with the MAPK pathway inhibitor PD98059 at a concentration of 25 µM, alone or together with the H_2_S donor GYY4137 at 10 µM. β-actin was used as a control for the total protein, and Na^+^/K^+^ ATPase α1 as a control for plasma membrane proteins. The β-actin and Na^+^/K^+^ ATPase bands shown in (A) were from the same sample, but not in all cases from the same lane on the gel, as the bands shown above or below. (**B–E**) The quantification of the bands and normalization of the signals was carried out as described in the legend to Figure 5. (B–F) Cells were exposed during the indicated times with 25 µM PD98059 (PD, black symbols) or with 25 µM PD98059 and 10 µM GYY4137 (GYY+PD, blue symbols). Comparison of the conditions by multiple Mann–Whitney tests did not reveal any significant difference. (B) Total ASIC1a expression, *n* = 4–5. (C) Plasma membrane ASIC1a expression, *n* = 4–5. (D) Erk1/2 expression, *n* = 4–5. (E) p-Erk1/2 expression, *n* = 4–5. (**F** and **G**) The current measurements were carried out in cultured hypothalamus neurons, as described in the legend to Figure 4. (F) pH 6.6-induced current amplitudes measured at the indicated times after the start of incubation with 25 µM PD98059 (PD, black) or with 25 µM PD98059 and 10 µM GYY4137 (GYY+PD, blue), *n* = 5–9. Together with each treatment condition, a number of control cells (ie, treatment protocol with solution lacking the H_2_S donor) were measured, and the current amplitudes obtained for the treatment and for the respective control were normalized to the average of the control. (G) pH 6.6-induced current amplitudes in cultured hypothalamus neurons obtained 24 h after the start of the incubation at the four following conditions: Control (culture medium), GYY (10 µM GYY4137), GYY+PD (25 µM PD98059 and 10 µM GYY4137), PD (25 µM PD98059), *n* = 7–8. *P < 0.05; **P < 0.01, between conditions, by one-way ANOVA test and Dunnett’s *post hoc* test. The current amplitudes were normalized to the mean amplitude of the control condition.

If the activation of the MAPK-Erk1/2 cascade is required for the H_2_S-induced increase in ASIC activity, the inhibition of the pathway should prevent the ASIC1a modulation by H_2_S. In a first experiment, the effect of the MAPK-Erk1/2 signaling pathway antagonist PD98059^76^^,^^77^ at 25 μM on H_2_S regulation of ASIC1a expression was tested in cultured primary cortical neurons. Neuronal cultures were incubated for the indicated times with either 25 μM PD98059 alone or with 25 μM PD98059 and 10 µM GYY4137. The GYY4137-induced increase of the total and plasma membrane ASIC1a expression (Figure 5A–C) was abolished by PD98059 (Figure 7A–C). PD98059 did not change the expression of Erk1/2 and prevented the increase of the intensity of the p-Erk1/2 bands (Figure 7D and E). The potentiation of the pH 6.6-induced current by GYY4137 in cultured hypothalamus neurons (Figure 4C) was prevented by PD98059 at all time points tested (Figure 7F). The pH 6.6-induced current was also measured at the time point 24 h in control condition (no drug added), with GYY4137 alone and together with PD98059, and with PD98059 alone (Figure 7G), showing that PD98059 inhibited the GYY4137-induced current increase, but not the basal ASIC current. Taken together, our findings indicate that H_2_S potentiates ASIC currents *via* the MAPK-Erk1/2 signaling pathway, by an increased total and plasma membrane expression.

## Discussion

We show here that H_2_S donors increase currents of recombinantly expressed ASICs and of endogenous ASICs in cultured brain neurons. The current increases over time and stays increased for many hours. The potentiation of the current amplitude is paralleled by an increased total and cell surface expression. We show that exposure to H_2_S donors increases Erk1/2 signaling, and that pharmacological inhibition of the MAPK-Erk1/2 pathway prevents the H_2_S-induced increase in ASIC expression and current amplitude.

### Concentration Dependence of ASIC Regulation by H_2_S Donors

Although there are some controversies regarding the determination of biological H_2_S levels, it is generally estimated that in physiological conditions, mammalian cells and tissues are exposed to low micromolar H_2_S concentrations.^78^ The H_2_S levels are dynamically regulated and can therefore change rapidly. In one study, a free H_2_S concentration of ∼0.03 µmol·g^-1^ protein (estimated to correspond to ∼3 µM) was determined in brain tissue samples.^79^ Kun Qu et al. measured the sulfide pool (both free H_2_S and sulfane sulfur) concentration in brain tissue samples as ∼12 µM in control and ∼25 µM in an ischemic stroke mouse model.^69^ In our experiments with recombinant ASICs, a unique, short (40 s) exposure of NaHS at 1 mM was tested, which is much higher than the physiological concentrations. On recombinant ASIC1a, different NaHS concentrations were tested. A potentiation occurred at ≥ 30 µM NaHS and was maximal at a concentration of 1 mM. It was however not possible to establish a clear concentration dependence. Exposure to 3 mM NaHS induced a maximal potentiation at ∼12–30 min after the exposure, which decreased subsequently with time. NaHS is a salt that dissociates rapidly to yield H_2_S (as dissolved H_2_S and dissociated HS^−^).^78^ In our study, the NaHS concentration inducing a potentiation of ASIC currents was, with 30 µM, higher than the physiological concentrations. With a prolonged or repeated administration of NaHS, lower concentrations might have induced potentiation of ASIC activity. We have measured the effects of a prolonged release of H_2_S on ASIC function in experiments involving exposure of cultured hypothalamus neurons to the slow-releasing H_2_S donor GYY4137, which induced ASIC current potentiation at concentrations as low as 10 µM. It has been shown that H_2_S concentrations reached by GYY4137 are < 10% of the administered GYY4137 concentration,^56^^,^^80^ indicating that in these experiments, concentrations of <1 µM H_2_S potentiated ASIC currents.

In cultured hypothalamus neurons, the potentiation occurred at concentrations of 30–300 µM NaHS or 10–30 µM GYY4137, while in CHO cells, 1 mM NaHS also induced a potentiation of ASIC1a currents, and showed even a tendency towards an increased potentiation. This difference may be due to different ASIC subtypes in these cell systems, since CNS neurons express besides homotrimeric ASIC1a also heterotrimers containing ASIC1a together with ASIC2a or -2b, or it may be influenced by differential expression of the signaling pathways involved in this regulation. Besides, the decreased ASIC current potentiation at high H_2_S donor concentrations in neurons may be due to cell toxicity. While H_2_S has a protective effect on neurons at low concentrations,^81^ H_2_S donors have been shown to induce at higher concentrations cell death in a process that involves glutamate receptors.^82^^,^^83^ This toxicity may prevent ASIC potentiation. CHO cells are more resistant to the H_2_S toxicity because they do not express glutamate receptors.

### H_2_S Inhibition of ENaC and ASIC

As mentioned in the introduction, H_2_S is known to regulate many different ion channels, among them the closely related ENaC. It was shown that ENaC activation by different means was prevented by NaHS in a distal nephron cell line,^84^^,^^85^ indicating that H_2_S has an inhibitory effect on ENaC. A related study showed that dexamethasone inhibits H_2_S-induced pulmonary edema in rats by preventing H_2_S-induced downregulation of α-ENaC.^86^ Very recently, NaHS-induced potentiation of ASIC1a, -2a, and -3, recombinantly expressed in CHO cells, was described.^63^ These authors observed a potentiation of ASIC currents after a 3- to 5-min exposure to 200 µM NaHS. In contrast to our data, the potentiation was rapidly reversible. It appears however that the authors did not continue the experiment for as long as we did in our study. This previous study was limited to recombinant ASICs. It did not provide any information on possibly involved signaling pathways or mechanisms, besides a conclusion that extracellular Cys residues on ASICs are not involved.^63^

### H_2_S May Not Act Directly on ASIC1a

The molecular mechanism by which H_2_S exerts its action involves the modification of Cys residues by S-sulfuration (or persulfidation), and this modification may cause functional changes in conformations, activities, and subcellular localization of the target proteins.^87^ H_2_S reacts with various molecules to create a mixture of biologically active species (polysulfides, persulfides).^78^^,^^88^ In addition, interactions between H_2_S and NO generate several potential intermediates.^78^^,^^89^ These species induce S-sulfuration of Cys residues in the target proteins.^78^^,^^90^^,^^91^ Cysteine S-nitrosylation and S-sulfination are endogenously occurring post-translational modifications of proteins. Such S-nitrosylated or S-sulfinated Cys residues can be S-sulfurated by H_2_S.^92^^,^^93^

In the CNS, ASIC1a is the most prominently expressed ASIC subunit. According to the structural models, human ASIC1a has one extracellular unpaired Cys residue, Cys275, which is located in the palm.^62^ Besides, there are three Cys residues in the TM1 domain (Cys49, Cys59, and Cys61) and four in the intracellular C-terminus (Cys466, Cys471, Cys497, and Cys528). For the regulation of ASIC1a by redox reagents, it was concluded that Cys61 is involved in the effects of oxidizing reagents, whereas Lys133 appeared to be involved in the actions of reducing agents.^32^ This regulation of ASICs by redox reagents is transient, and affects only ASIC1a but not other ASICs,^32^ strongly suggesting that its mechanism is different from that of the regulation by H_2_S. One study highlighted the importance of intracellular C-terminal Cys residues for the inhibition of ASIC1a currents by millimolar concentrations of the oxidant H_2_O_2_, showing that H_2_O_2_ induces the formation of intersubunit disulfide bonds.^94^ By testing the mutant ASIC1a-ΔCCt in which the four C-terminal Cys residues are mutated or removed, we found no evidence for an involvement of these Cys residues in the modulation of ASIC1a by H_2_S. Although directly after NaHS exposure, the current was increased in the WT but not in the mutant, this difference was not statistically significant. The cited study on ASIC modulation by NaHS concluded that H_2_S does not modify extracellular Cys residues of ASIC1a.^63^ Although the Cys residues of the TM1 have not been tested, it appears likely that the H_2_S donors do not induce a modification of ASIC1a Cys residues and may rather affect signaling pathways that affect ASIC function and expression.

### Time Dependence of the ASIC Current Increase in Cultured Hypothalamus Neurons

Exposure of CHO cells expressing ASIC1a to 1 mM NaHS during 40 s induced an ASIC current potentiation that was measurable directly after the NaHS exposure and further increased during the ∼1 h of the measurement. This suggests that there is a direct regulatory component of the H_2_S effect, but that H_2_S affects in addition the expression and/or the ASIC trafficking. The ASIC current modulation by H_2_S donors was followed over a longer time period in cultured hypothalamus neurons. Both, NaHS and GYY4137 potentiated the ASIC currents 1 h after exposure, and at different time points, up to 24 h for NaHS and 72 h for GYY4137 after exposure, not however at 2 h after exposure. The biochemical analysis indicated a significant increase in total ASIC1a expression at ≥8 h after GYY4137 exposure. The increase of ASIC1a expression at the plasma membrane was significant at 1 h, and at ≥4 h after GYY4137 exposure. Many ion channels, such as AMPA receptors and ASIC1a, undergo both constitutive and regulated endocytosis, which act cooperatively to achieve homeostasis and/or plasticity in response to different environmental changes.^95^^,^^96^ Accumulation of ASIC1a in the plasma membrane can induce constitutive endocytosis in a clathrin- and dynamin-dependent manner in cortical neurons.^96^ A possible underlying mechanism may be the following. The increase in cell surface expression and current amplitudes observed at 1 h after exposure to H_2_S donors may be mostly induced by increased trafficking of ASIC1a to the plasma membrane. The net increase in cell surface expression may be transiently stopped by increased endocytosis (having the strongest effect at 2 h), which would then only be overcome after the increase in ASIC1a expression that takes more time to develop. This is consistent with the observation that H_2_S regulates not only trafficking but also the expression of ASIC1a.

### Several Signaling Pathways Are Involved in the Regulation of ASIC Expression

Although there is a small, immediate increase in ASIC currents after exposure to NaHS (Supplementary Figure S2), it appears that the large part of the current increase takes longer to develop. Several signaling pathways are known to participate in the regulation of ASIC trafficking and expression, such as protein kinase A (PKA),^97^^,^^98^ protein kinase C (PKC),^99^ the phosphoinositide 3-kinase-protein kinase B (PI_3_K-AKT), and extracellular signal-regulated kinase 1/2 (Erk1/2).^74^ Erk1/2 belongs to the family of MAPKs, which are protein Ser/Thr kinases that respond to a wide range of extracellular stimuli.^75^ Three major mammalian MAPKs, ERK1/2, JNK, and p38 kinase, are regulated by distinct signal transduction pathways that control many aspects of mammalian cellular physiology.

H_2_O_2_ at a concentration of 20 µM was shown to upregulate ASIC1a expression through the MAPK-JNK signaling pathway in NS20Y cells and primary cultures of cortical neurons.^100^ In cultured spinal dorsal horn neurons, activation of the PI_3_K-AKT-Erk1/2 cascade enhanced ASIC1a currents *via* phosphorylation of the cytoplasmic residue Ser25 of ASIC1a, resulting in enhanced forward trafficking and increased surface expression.^74^ Activation of PKC increased ASIC1a protein expression and ASIC currents in cultured cortical neurons, and PKC regulation of ASIC1a protein expression involves the NF-κB signaling pathway.^99^^,^^101^ p-Erk1/2 regulates not only the trafficking but also the expression of ASICs; activation of Erk1/2 enhanced forward trafficking in cultured spinal dorsal horn neurons.^74^ p-Erk1/2 can lead to activation of NF-κB, which in turn was shown to regulate the transcriptional expression of ASICs.^99^^,^^102^

There is evidence that H_2_S can activate several signaling pathways. NaHS was shown in transfected HEK-293 cells and in rat vascular smooth muscle cells to increase phosphorylation of Erk1/2 and of PKC.^103^ In isolated rat hearts, H_2_S stimulated both cardiac Akt and PKC activity.^104^ In the context of ENaC inhibition in H_2_S-induced pulmonary edema in rats, H_2_S induced Erk1/2 expression and phosphorylation.^86^ The mechanisms for H_2_S-induced MAPK signaling activity are complex and likely depend on the cell type and on the concentrations used.^105^

In the present study, exposure of cultured cortical neurons to 10 µM GYY4137 did not change the expression of Erk1/2; however, it increased the phosphorylation of Erk1/2, indicating that it activated the pathway. In contrast, 10 µM GYY4137 did not activate the JNK and p38 signaling pathways. The Erk1/2 pathway inhibitor PD98059 prevented the GYY4137-induced increase in ASIC1a expression in cultured cortical neurons and the GYY4137-induced increase in ASIC currents of cultured hypothalamus neurons, indicating that the activation of the Erk1/2 pathway is required for the H_2_S-induced ASIC current increase.

It is known that H_2_S, NO, and reactive oxygen species (ROS) interact with each other in their production, downstream signaling, and by direct chemical interaction, and this in different organs.^78^^,^^106^^,^^107^ In rat neonatal cardiomyocytes, H_2_S inhibits mitochondrial complex IV and activates superoxide dismutase to decrease the levels of ROS in cardiomyocytes during ischemia/reperfusion.^108^ H_2_O_2_, a major ROS, upregulates ASIC1a expression through the MAPK–JNK signaling pathway in NS20Y cells and primary cultures of cortical neurons.^100^ In our study, we did however not detect an activation of JNK by GY4137. H_2_S has been shown to increase NO levels in some tissues. Interestingly, NO potentiates ASIC currents, and there is evidence that this regulation involves direct oxidation of Cys residues.^33^ Our study strongly suggests an indirect regulation of ASICs by H_2_S, and currently, there is no evidence for an interplay between these gasotransmitters in the regulation of ASIC activity.

In this work, we have characterized the regulation of ASICs by exogenous H_2_S. To examine whether endogenous H_2_S can exert such a regulation, future experiments will use silencing or pharmacological inhibition of the enzymes that produce H_2_S. Silencing of CSE with siRNA, or pharmacological inhibition of this enzyme both decreased the activation of Erk1/2,^109^ while the overexpression of CSE increased the activation of Erk1/2.^110^ These observations are consistent with a possible effect of endogenous H_2_S on ASICs.

### Possible Physiological Importance of ASIC Regulation by H_2_S

ASICs detect tissue acidosis occurring upon tissue injury, inflammation, ischemia, stroke, and tumors as well as fatiguing muscle, to activate pain-sensing nerves in the periphery and transmit pain signals to the brain. ASIC1a was shown to protect against seizures by shortening their duration,^17^ and ASIC1a activation is also involved in synaptic plasticity, learning, and memory.^11^ Dysfunction of ASIC1a may contribute to the learning and memory deficit associated with Alzheimer’s disease.^111^^,^^112^ ASIC2 is a negative modulator of rod phototransduction, and functional ASIC2 channels are beneficial for the maintenance of retinal integrity.^113^ H_2_S can improve the hippocampal damage induced by recurrent febrile seizures,^114^ and protect the retina in the context of retinal vascular diseases.^115^ H_2_S is also involved in the regulation of neural synaptic plasticity and cognition^116^ and it attenuates spatial memory impairment and hippocampal neuroinflammation in the Aβ1 rat model of Alzheimer’s disease.^117^ The mechanism of the function of H_2_S in these processes is still unclear, and it is possible that regulation of ASICs by H_2_S may be involved.

Taken together, we found that H_2_S potentiates ASIC currents in a time- and concentration-dependent way. This potentiation does not depend on the acid sensitivity of ASIC1a but is induced by an increased expression of ASIC1a at the plasma membrane. Our data suggest that this regulation, which is likely of importance in several physiological and pathological conditions, is mediated by the MAPK-Erk1/2 signaling pathway.

## Supplementary Material

zqab007_Supplementary_Data
