# Supplementary material for: Hydrogen Sulfide Upregulates Acid-sensing Ion Channels via the MAPK-Erk1/2 Signaling Pathway
Source: Function (Oxf). 2021 Feb 19;2(2):zqab007. doi: 10.1093/function/zqab007 (PMC8833866; doi:10.1093/function/zqab007)
Supplement: zqab007_Supplementary_Data [file zqab007_Supplementary_Data.docx]

**Supplementary Figures for "Hydrogen sulfide upregulates acid-sensing ion channels *via* the MAPK-Erk1/2 signaling pathway"**

Zhong Peng, Stephan Kellenberger


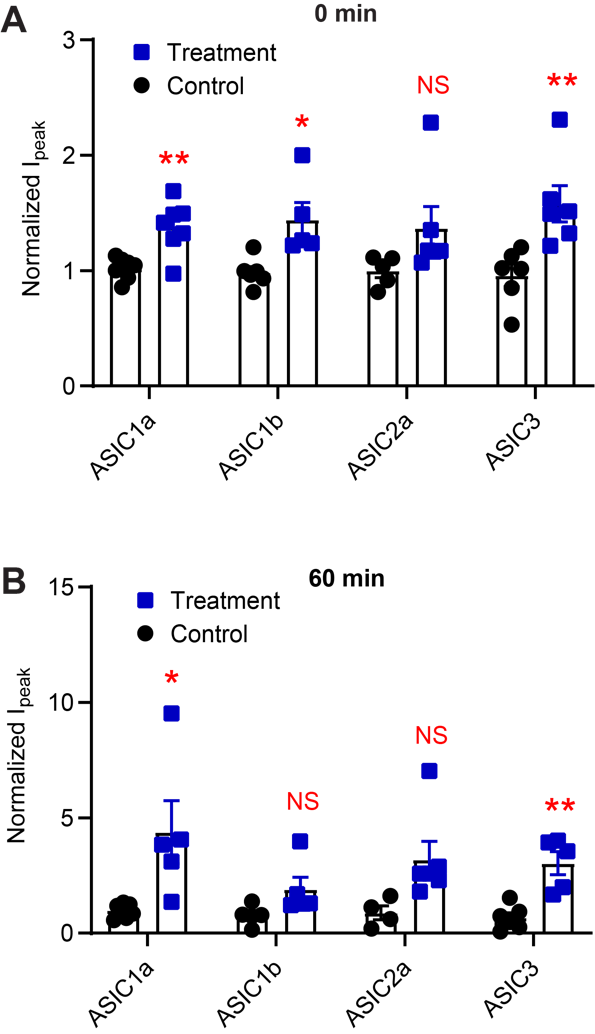


## Figure S1. Peak current amplitudes of homomeric ASIC currents immediately after and 60 min after NaHS exposure. A, Normalized peak current amplitudes of different ASIC isoforms expressed in CHO cells immediately after a 40-s exposure to 1mM NaHS (Treatment, blue) or to a control solution (Control, black), n=5-7. B, Same experiment as in A, but at time point 60min after NaHS exposure. In all experiments, the currents were normalized to the amplitudes measured before NaHS (or control) exposure (at -3 and -6 min). *, p<0.05; **, p<0.01, comparison with the corresponding control experiments, Multiple Mann-Whitney tests.


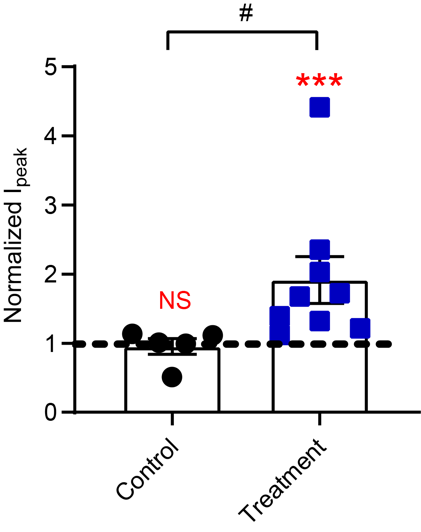


## Figure S2. Proof of ASIC current potentiation in cells used for the analysis of the pH dependence. Current measurements of ASIC1a-expressing CHO cells used for the analysis of the pH dependence (Figure 3A-D). The pH6.7-induced peak current amplitudes, measured at 15 min after a 40-s 1mM NaHS or control solution exposure were normalized to the pH6.7-induced peak current amplitude measured before the NaHS (Treatment, blue symbols) or control exposure (Control, black symbols) in the same cell. ***, p<0.001, current increase compared with the current amplitude before NaHS exposure in the same cells (dash line), paired Student’s *t*-test. #, p<0.05, current increase different in NaHS compared to control group, by unpaired Student’s *t*-test.


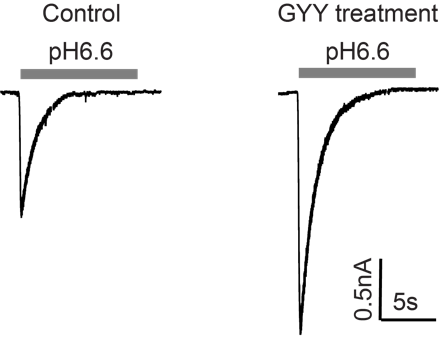


## Figure S3. Typical acid-induced current traces in cultured hypothalamus neurons. Representative current traces in cultured hypothalamus neurons induced by pH6.6 with or without 10uM GYY4137 treatment as indicated, recorded with whole-cell patch-clamp at -60 mV.
